# Fournier gangrene – would you KISS it?

**DOI:** 10.3205/iprs000182

**Published:** 2023-12-11

**Authors:** Miguel João Ribeiro Matias, Diogo Guimarães, Manuel Vilela, Juliana Sousa, Joaquim Bexiga

**Affiliations:** 1 Centro Hospitalar Universitário Lisboa Central, Lisbon, Portugal

**Keywords:** Fournier gangrene, pudendal flap, skin graft, loco regional flaps, perineal reconstruction

## Abstract

Fournier gangrene is a disease characterized by necrotizing fasciitis of the perineal and genital region, resulting from synergistic polymicrobiotic infection. Most infections can be localized to a cutaneous, urethral, or rectal source and can culminate in a fulminant sepsis. Current state of the art is systemic broad-spectrum antibiotics and serial aggressive debridement which result in superficial perineal defect of wide dimensions.

We compiled all the cases of Fournier gangrene that required reconstruction after debridement in Centro Hospitalar Universitário Lisboa Central from 2018 to 2022. Inclusion criteria were reconstruction for Fournier defects and patients’ age 18 to 90 years old. Exclusion criteria were patients who didn’t require reconstruction or didn’t complete it due to death or transfer to another healthcare institution. Reconstructive procedures and complication rates are reported as whole numbers and percentages of total.

The initial search yielded 32 patients. There were 2 (6.2%) patients with defects that healed by secondary intention, 6 (18.7%) with delayed primary closure, 4 (12.5%) with implantation of the testicle in a medial thigh pocket, 12 (37.5%) with skin grafts, 4 (12.5%) with scrotal advancement flaps, 2 (6.2%) with flaps, and 2 (6.2%) with flaps and skin grafts in combination. Four outcomes were evaluated: number of patients, defect size, method of reconstruction, and wound-healing complications.

Most reconstructive techniques provide reliable coverage and protection of testicular function with an acceptable cosmetic result. The reconstructive options need to be patient tailored in order to achieve long lasting results with a minimum of postoperative morbidity.

## Introduction

Fournier gangrene is a serious, life-threatening infection of the genital and perineal areas, which can rapidly spread to surrounding tissue and organs [1], [2], [3], [4], [5]. It is a type of necrotizing fasciitis, which is a rare but severe bacterial infection that destroys skin, fat, and tissue covering the muscles, as well as the muscles themselves [1], [2], [3], [4], [5].

Fournier gangrene most commonly affects men, although women and children can also be affected [6]. The exact incidence and prevalence of Fournier gangrene are difficult to determine as the condition is often underdiagnosed and underreported. Risk factors associated with the disease are:

- diabetes mellitus: the most commonly identified risk factor for Fournier gangrene, and patients with poorly controlled diabetes are at higher risk for developing the condition [2],
- immunocompromised state: patients with HIV/AIDS, cancer, or organ transplant recipients,
- alcoholism,
- obesity and
- recent trauma or surgery.

The most common microorganisms that are associated with Fournier gangrene include various types of bacteria, including both aerobic and anaerobic species [7]. Some of the most frequently isolated microorganisms from patients with Fournier gangrene include *Escherichia coli*, *Klebsiella pneumoniae*, *Bacteroides fragilis*, *Peptostreptococcus* species, and *Streptococcus* species [7].

In addition to these microorganisms, other bacteria and fungi may also be implicated in some cases of Fournier gangrene. The specific microorganisms that are present may vary depending on a variety of factors, such as the patient’s immune status, underlying health conditions, and previous exposure to antibiotics or other antimicrobial agents [7].

Symptoms of Fournier gangrene include severe pain and swelling in the affected area, along with redness, warmth, and tenderness. The skin may also appear shiny, taut, and discoloured, or with the presence of bullae. Other symptoms may include fever, chills, and rapid heart rate [2], [3]. Symptoms may progress rapidly, and Fournier gangrene is considered a medical emergency that requires prompt diagnosis and treatment [4].

Fournier gangrene is a devastating condition that requires prompt and aggressive treatment to prevent morbidity and mortality. Non-surgical treatments for Fournier gangrene include broad-spectrum antibiotics, which are replaced by targeted antibiotics based on bacterial cultures and sensitivities. Topical antiseptics or wound dressings may be used to reduce bacterial load and promote granulation tissue formation. Adequate nutritional support and regular dressing changes are also crucial in promoting healing [8].

Hyperbaric oxygen therapy (HBOT) is another non-surgical treatment option that may be used to control bacterial growth and promote tissue healing. HBOT involves the use of high-pressure oxygen to improve oxygen delivery to tissues [9].

It is important to note that non-surgical treatment options are typically used in combination with surgical intervention and are not considered to be stand-alone treatments for Fournier gangrene. Prompt diagnosis and early treatment with a multidisciplinary approach are crucial in improving outcomes and reducing the risk of complications. Further research is needed to optimize the use of non-surgical treatments in the management of Fournier gangrene.

Surgical intervention, including debridement and drainage of necrotic tissue, is the primary treatment modality. However, non-surgical treatments may be used in conjunction with surgical intervention or in situations where surgery is not possible [8].

The extent of surgical intervention required depends on the severity and extent of the infection [10]. The initial step in surgical treatment is to establish adequate drainage of purulent material and to remove any visible necrotic tissue. This is usually accomplished through wide incisions in the affected area, with subsequent removal of all nonviable tissue, including fascia and muscle if necessary. The goal of surgery is to achieve healthy wound edges and promote granulation tissue formation.

In some cases, more extensive surgical procedures may be necessary, such as colostomy or cystostomy for faecal or urinary diversion, respectively. 

Postoperatively, patients are closely monitored for signs of ongoing infection, and antibiotics are continued until the infection is well-controlled. Additional surgical debridement may be necessary if signs of ongoing infection persist.

Reconstruction following Fournier gangrene is a challenging process that is dependent on the extent and severity of tissue damage and the overall health status of the patient. While spontaneous healing may occur in some cases, reconstructive surgery is often necessary to achieve optimal outcomes [2], [8].

Reconstructive options include skin grafting, flap reconstruction, and tissue expansion [3], [5], [8], [11]. Skin grafting involves transplanting healthy skin from another area of the body to the affected site. Flap reconstruction involves transferring a segment of healthy tissue with its blood supply to the wound bed. Tissue expansion involves gradually stretching healthy skin to create new tissue to cover the wound. Free flap reconstruction is typically not needed [10].

In cases where the infection has spread to the abdominal cavity or pelvic area, reconstruction may require the use of mesh or other materials to provide support to the abdominal or pelvic organs.

The optimal timing for reconstructive surgery is after the infection has been controlled and the wound is free of infection. The timing of reconstruction depends on the extent of tissue damage and the patient’s overall health status [6].

It is essential to note that reconstructive surgery following Fournier gangrene is a complex process that requires a multidisciplinary approach involving plastic surgeons, urologists, and general surgeons. Close monitoring and follow-up are also necessary to ensure optimal outcomes and detect any potential complications.

## Methods

We compiled all the cases of Fournier gangrene that required reconstruction after debridement in Centro Hospitalar Universitário Lisboa Central from 2018 to 2022. Inclusion criteria were reconstruction for Fournier defects and patients’ age 18 to 90 years old. Exclusion criteria were patients who didn’t require reconstruction or didn’t complete it due to death or transfer to another healthcare institution. We accessed the following variables: defect size, location, laterality, method of reconstruction, complications and basic epidemiologic data such as sex, age and presence of co-morbidities.

Reconstructive procedures and complication rates are reported as whole numbers and percentages of total.

## Results

Our cohort had 32 patients (n=32). All patients were male (n=32; 100%). The age distribution is shown in Table 1 (Tab. 1). There were no patients under the age of 20. The cohort of highest age was between 50 and 60 years old with 12 patients (n=12; 37.5%).

The most common co-morbidities in our cohort were hypertension, obesity (IMC>30), diabetes, and dyslipidemia. Notable comorbidities were the presence of inflammatory bowel disease, chronic kidney disease and HIV (Table 2 (Tab. 2)). The most common predisposing factors were diabetes (n=12), inflammatory bowel disease (n=6), chronic kidney infection (n=2) and HIV (n=1) (Table 3 (Tab. 3)). Eleven patients presented with no known predisposing factor.

The most common microorganism isolated was *Staphylococcus aureus* (n=14; 43.8%), followed by *Enterococcus* faecalis (n=6, 18.8%) (Table 4 (Tab. 4)). The most common gram-negative microorganism was *Bacteroides fragilis* (n=4).

In our cohort, cause of the infection was idiopathic in 17 patients (Table 5 (Tab. 5)). The most common causes were perineal abcess (n=6), skin abcess (n=5) and perineal trauma (n=4). Cultures were obtained in all initial debridements and the isolated microorganisms are shown in Table 4 (Tab. 4).

In our study, we defined laterality as unilateral, if it did not pass the midline of the perineum, and bilateral if it did. Twenty-four patients had unilateral disease, and 8 patients had bilateral disease. We divided the perineal involvement into scrotal, penile or penoscrotal. Twenty-eight patients had scrotal involvement and 4 had penoscrotal involvement. There were no cases of isolated penile involvement.

We divided the area in over or under 50 cm². This threshold was based on the current literature. In our study, 19 patients had an area of involvement >50 cm² and 13 <50 cm².

The methods of reconstruction were classified as delayed primary intention, secondary intention, skin grafting, testicle pocketing, local flaps and local flaps with skin grafting. The results are shown in Table 6 (Tab. 6).

The following complications were observed in our cohort (Table 7 (Tab. 7)): In the local flap subgroup, there were 2 wound dehiscences and 2 partial flap losses. In the testicle pocketing subgroup, there were 2 cases of wound dehiscence. In the skin grafting subgroup there were 4 cases of partial graft digestion. In the local flap and skin grafting subgroup there was one case of wound dehiscence and one case of partial flap loss.

## Discussion

Fournier gangrene is characterized by a necrotizing infection that is rapidly progressing and potentially fatal. It starts with the inoculation of multiple microorganisms in the soft tissues of the external genitalia and perineum extending along the Bucks fascia of the penis, Colles fascia of the perineum and Scarpas fascia of the abdomen [1], [2]. This progression along the fascial plane leads to oedema, vessel dissection and microthrombosis, leading to localized hypoxia and promoting anaerobic microorganism overgrowth [4]. 

In our cohort, the most common gram-negative microorganism was the *Bacteroides fragilis* (n=4). However, most wound cultures performed were with a swab and tested for aerobic organism in the initial stages of the disease which represents a source of bias in the sample. The most common microorganism isolated was *Staphylococcus aureus* (n=14; 43.8%), followed by *Enterococcus faecalis* (n=6, 18.8%), which represent the most common microorganism in the skin and lower gut flora.

Most cases of Fournier gangrene require a mixture of factors: a predisposing host with a source of infection [2]. The most common predisposing factor was Diabetes Mellitus (n=12), whose effects on the immune system are widely known. The second most common predisposing factor was the presence of an inflammatory bowel disease. This entity is known for an altered gut microbiome, along with the predisposition for anal fissuring, abscesses and perianal lesions. Most patients are also under immunosuppressive medication, which further increases the risk of a polymicrobial infection. Other known predisposing factors were the presence of chronic kidney disease (n=2) and HIV (n=1), which are also associated with an impaired immune system. 

There is ample literature regarding the reconstructive options available for the perineal and scrotal region after soft tissue loss [10]. Fournier gangrene is classified as a superficial soft tissue defect, sparing the pelvic organs. However, in the process of surgical debridement or contiguous abscess formation, the pelvic muscles and the reproductive system can be affected. Patients should be subjected to aggressive debridement and prompt coverage of the soft tissue defect, aiming for the most accessible and speedy reconstructive method.

In our cohort, 28 patients had scrotal involvement and 4 had penoscrotal involvement. When the penis is involved, the use of skin grafts is the main treatment option. The use of split thickness skin graft was the reconstructive option of choice. If under 50% of the testicle is exposed, the use of testicle pocketing is a valid option. However, for exposures >50% of the testicle of bilateral exposure, the use of locoregional flaps is the main treatment option. We classified the area of total perineal and abdominal involvement using the 50 cm² cut off. This cut off was arbitrarily defined in order to give a perspective of the size of the soft tissue defect. Nineteen patients had a defect that was over 50 cm² and 13 had an area under 50 cm². This sample is biased because most small defects were managed conservatively in the Urology and General Surgery Department, only when defects were bigger or had exposition of the testis, they were sent to the Plastic Surgery Department. Another handicap was the fact that the referral to the tertiary center sometimes was done weeks after the last debridement, which present wounds in a more advanced stage of healing. Due to this fact, 2 (6.2%) patients were closed by secondary healing, with regular dressing changes and vacuum assisted wound closure and 6 (18.8%) patients were closed by delayed primary intention. In those who had exposed testes, 4 (12.5%) were submitted to testicle pocketing, 6 (18.8%) were submitted to local flaps and 2 (6.2%) were submitted to local flaps and skin grafting. The most common used local flaps were rotational or island flaps based on the internal pudendal artery or the superficial circumflex artery. Twelve patients (37.5%) were submitted to skin grafts, mostly due to perineal defects not involving the scrotal area.

The delayed primary closure subgroup was submitted to surgery within one week in all cases. In the secondary intention healing subgroup, one case took 4 weeks and one 6 weeks to close fully. The split thickness skin graft had a partial loss in 33.3% of cases, which the author defined as the loss of more than 10% of the grafted area, and is compatible with the current literature. In the testicle pocketing subgroup, the flap subgroup and the flap and skin grafting subgroup there were cases of wound dehiscence. This can be explained due to the nature of the perineal area. Even though it is well vascularized, the perineal area is used for support of the internal organs during walking, being under constant stretch and is an essential linchpin for support when in decubitus or in Fowler’s position. Furthermore, most of these patients wear disposable diapers in order to facilitate wound care, which are often prone to increase the local humidity in the wound milieu. 

Comparing with published literature it is well recognized that most reconstructive solutions provide reliable cover and an acceptable cosmetic result. Karian et al.’s systematic review [12] of over 425 patients presented similar results to our study. 5.9% of patients healed by secondary intention, compared with 6.3% in our cohort (n=2); 10.4% were treated by delayed primary closure which is considerably lower than the rate of 18.7% in our cohort. This difference can be justified by the selection criteria of the articles of the systematic review, where some articles do not include patients that were treated by delayed primary closure. In our cohort, 12.5% of patients were submitted to testicle pocketing which is comparable to the 8.5% in the international literature. The skin graft cohort in our study represents 37.5% which is higher than 22.6% of the systematic review. Furthermore, our local flap rate was 18.7% compared with 30.1%. Two hypotheses may justify this difference. First, some patients who presented to the Plastic Reconstructive Surgery Department in our University Hospital were referred from district hospitals. The transfer of these patients is normally delayed until the debridement is complete and the patient is stabilized for transfer. These conditions may allow the wound to granulate under negative pressure therapy and allow for a granulated wound bed that is suitable for grafting. The second hypotheses that might justify this difference is the type of patients that present in our tertiary centre hospital. The cases are often more complex and the patients have a larger area to cover.

Regarding microbiological data, Tang et al. [13] accessed the trends in microbiology of Fournier gangrene in 22 studies published over 9.2 years with 4,365 reported cases. They reported the most common microorganism to be *Escherichia Coli* (46.6%), followed by Streptococcus (38.1%). As these studies accessed all published articles, without separating the geographic location, the prevalence and incidence of microorganism and drug resistance may be responsible for the difference in the results.

Epidemiological evidence for Fournier gangrene in Portugal is scarce, however, Louro et al. [10] have conducted a similar study in 2019 in Centro Hospitalar Universitário de Coimbra. The most prevalent microorganism found were *Staphylococcus aureus* (n=7; 46.7%); *Enterococcus faecalis* (n=5; 33.3%); *Escherichia Coli* (n=3; 20%). In our cohort, we had the same most common microorganisms with a similar distribution: *Staphylococcus aureus* (n=14; 43.8%); *Enterococcus faecalis* (n=6; 18.8%); *Escherichia Coli* (n=4; 12.5%). Interestingly, comparing with Louro et al.’s [10] cohort, our cohort presented no *Aspergilus fumigatus* but a higher incidence of *Bacteroides fragilis* (n=4; 12.5%).

In terms of complications, in our cohort, there were 3 cases of partial flap loss, which was defined as the presence of necrosis on a portion of the flap but without the reconstructive procedure being compromised. In the local flap group, two of the pudendal artery pedicled flaps suffered partial necrosis of the most distal portion of the flap. In the flap and skin graft subgroup one propeller pudendal perforator flap suffered distal flap necrosis due to excessive tension. All the partial flap necrosis healed with dressing changed and no further procedures were required.

## Conclusion

Fournier gangrene is a necrotizing infection of the perineal soft tissues and external reproductive organs. It is associated with immunocompromising co-morbidities. Its most common microorganism is gram-negative, however, gram-positive from the skin flora that are inoculated into deeper tissues can also be responsible for this condition. Treatment consists of antibiotics and radical debridement which often leaves a soft tissue defect that requires a reconstructive procedure. 

Currently, the main reconstructive options are split thickness skin grafts, randomized local advancement flaps and pedicled flaps based on the superficial inguinal or pudendal vessels. All of these options are valid and need to be patient tailored in order to achieve long lasting results with the minimum of postoperative morbidity.

Due to the nature of the perineal area, with increased moisture and fecal contaminants, the reconstructive process is often filled with complications. So, we propose that sometimes, the simpler solution might be the more elegant one. In other words, we propose the KISS approach – Keep it simple, surgeon!

Two important deciding factors need to be accessed: The size and the structures involved in the defect. We propose that whenever the testis is exposed the use of testicle pocketing in the medial or local flaps should be the first line of treatment. However, if the defect involves the penis, the posterior perineal triangle or the inguinal and hypogastric region, a skin graft often provides adequate cover and allows for a more predictable postoperative course.

**Figure 1:**
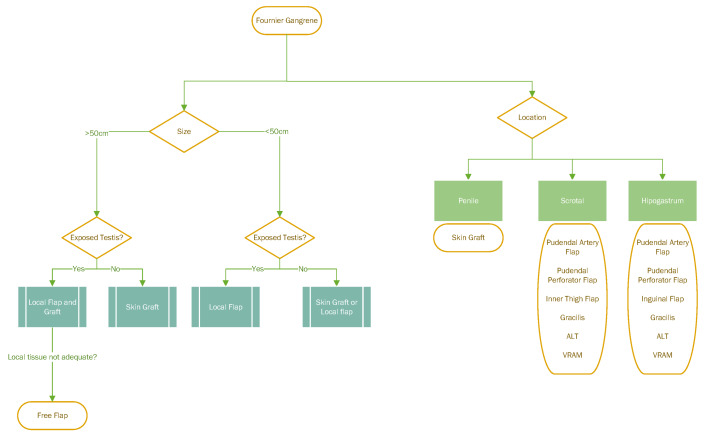
KISS approach

Multiple flaps can be used for different regions as shown in Figure 1 (Fig. 1). Our preference resides in the pudendal flaps or pudendal perforator flap due to its accessibility, the fact that it removes tissue from a less conspicuous area where there is normally redundant soft tissue and allows for direct closure.

## Notes

### Authors’ ORCIDs

- Miguel Matias: 0000-0002-2381-5603
- Diogo Guimarães: 0000-0003-2186-9816
- Manuel Vilela: 0000-0003-2186-9816
- Juliana Sousa: 0000-0002-3554-0598
- Joaquim Bexiga: 0009-0003-2864-3497

### Ethics

This study was performed in accordance with the Declaration of Helsinki. Ethics approval was obtained from the Lisbon Central Hospital Center Ethical Committee.

### Competing interests

The authors declare that they have no competing interests.

## Figures and Tables

**Table 1:**
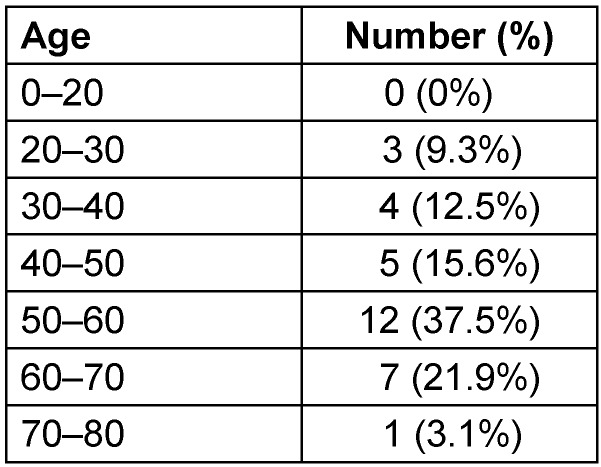
Age distribution

**Table 2:**
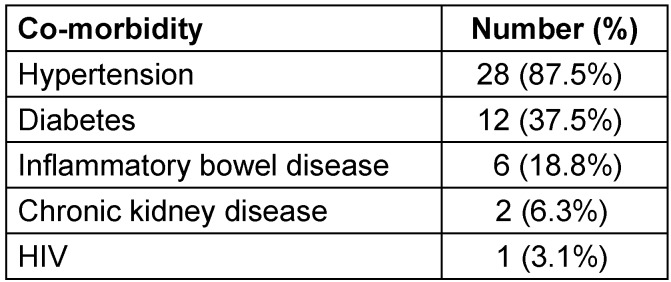
Co-morbidities

**Table 3:**
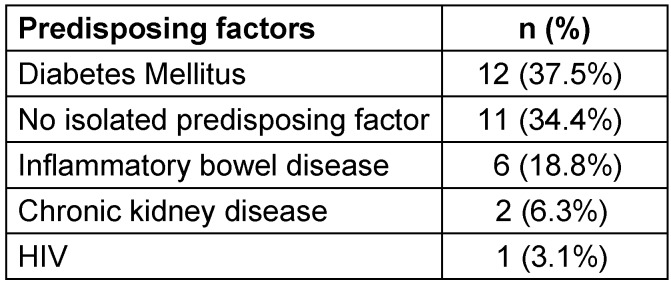
Predisposing factors

**Table 4:**
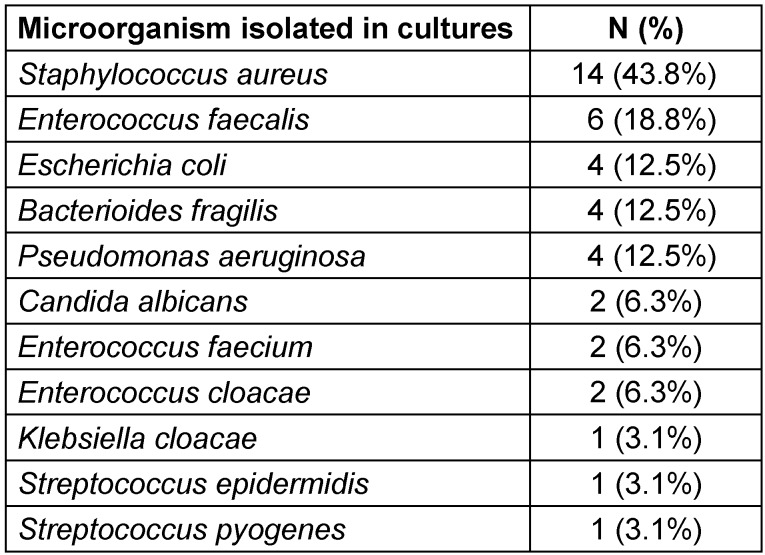
Isolated microorganisms

**Table 5:**
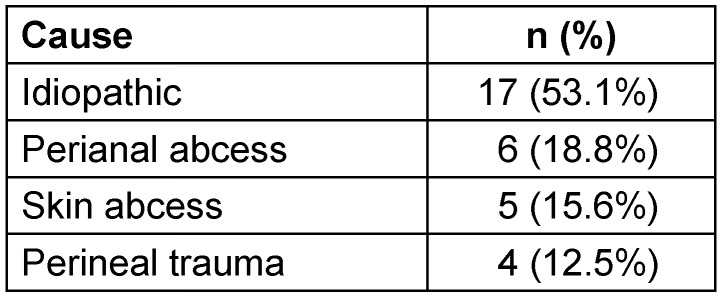
Causes

**Table 6:**
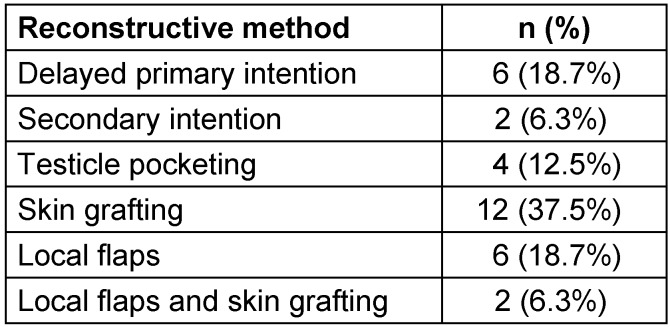
Methods of reconstruction

**Table 7:**
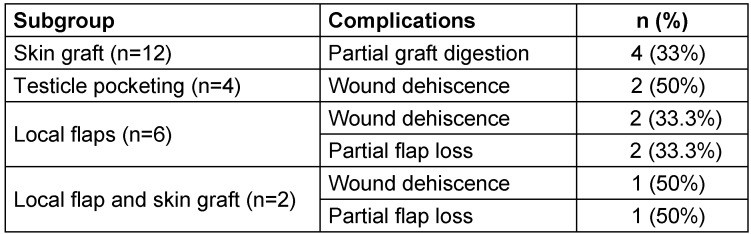
Complications
